# Spontaneous Tumor Lysis Syndrome due to Uterine Leiomyosarcoma with Lung Metastases

**DOI:** 10.1155/2017/4141287

**Published:** 2017-09-11

**Authors:** Vivek Alaigh, Debapriya Datta

**Affiliations:** ^1^Department of Medicine, University of CT Health Center, 263 Farmington Avenue, Farmington, CT 06030, USA; ^2^Division of Pulmonary-Critical Care Medicine, University of CT Health Center, Farmington, CT 06030, USA

## Abstract

Tumor lysis syndrome (TLS) is an oncologic emergency characterized by a combination of metabolic derangements (hyperuricemia, hyperkalemia, hyperphosphatemia, and hypocalcemia) caused by rapid turnover from cell destruction in certain cancers. These metabolic derangements can lead to seizures, cardiac arrhythmias, renal failure, and death. TLS is usually seen after the initiation of chemotherapy for hematologic malignancies. TLS occurring spontaneously, without initiation of chemotherapy, is rare and its occurrence in solid tumors is rarer still. We report a case of spontaneous TLS in a patient with leiomyosarcoma of the uterus, with metastasis to lung. Such a case has never been reported before.

## 1. Introduction

Tumor lysis syndrome (TLS) is an oncologic emergency resulting from lysis of tumor cells with release of intracellular contents into the circulation [1]. It consists of hyperuricemia, hyperkalemia, hyperphosphatemia, and hypocalcemia caused by rapid breakdown of tumor cells [2]. These derangements can lead to seizures, cardiac arrhythmias, renal failure, and death.

TLS is most commonly seen during the initiation of chemotherapy in aggressive hematologic malignancies, such as acute myeloid leukemia, acute lymphoblastic leukemia, and non-Hodgkin's lymphoma [3]. Less often, it has been seen in the treatment of solid bulky tumors that are sensitive to chemotherapy, such as breast cancer and small cell lung cancer [3]. Of note, there is not a systemic relationship between the size of a solid tumor and the occurrence of TLS. TLS occurring spontaneously without prior treatment is rare, though reported in hematologic malignancies. Even rarer is spontaneous TLS in solid tumors. Rarely does TLS occur spontaneously with solid tumors. There has not been any previously documented case of spontaneous TLS occurring in a uterine malignancy with lung metastases.

## 2. Case Description

**Figure 1:**
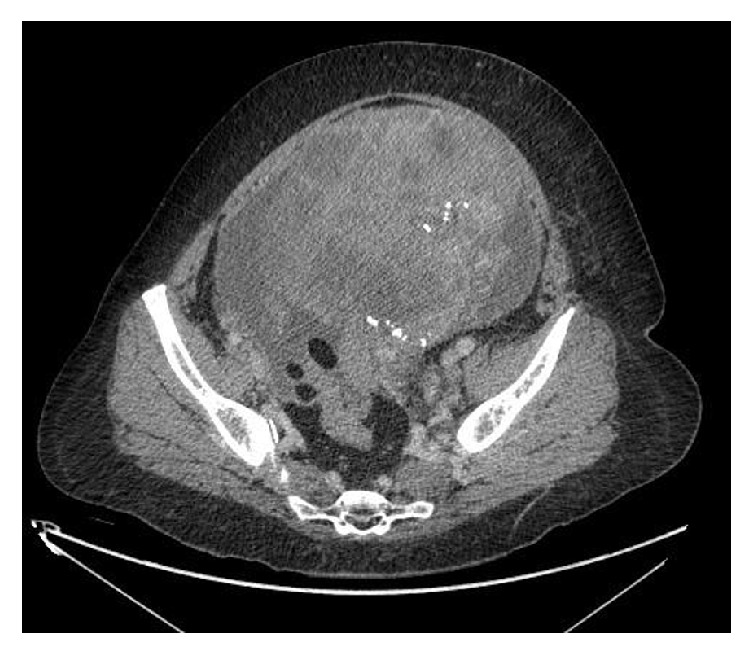
CT scan of the abdomen showing a large heterogeneous, partially necrotic abdominal-pelvic mass, 16.3 cm by 20.2 cm in size.

**Figure 2:**
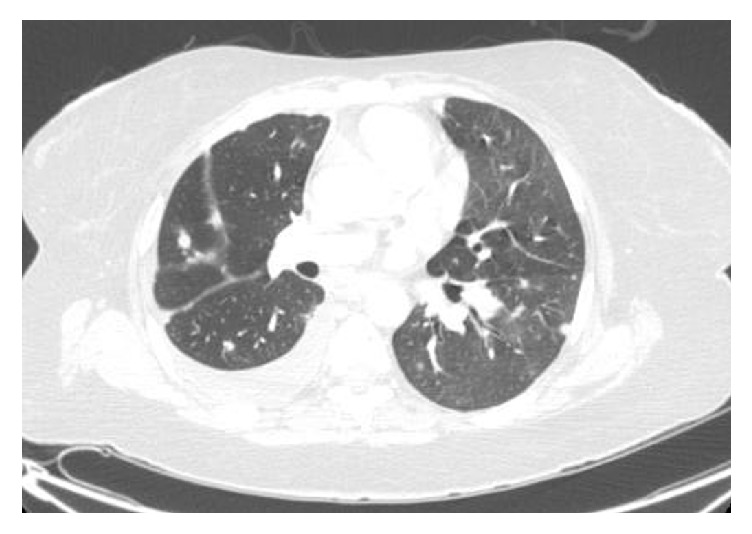
CT scan of chest showing a moderate right-sided pleural effusion, as well as bilateral pulmonary nodules with hilar adenopathy.

A 58-year-old Caucasian female with no significant past medical history presented from a community hospital with a 3-week history of abdominal distension and constipation. She also complained of nausea, fatigue, shortness of breath with exertion, and decreased appetite. On examination, the patient had an extremely firm and distended lower abdomen with tenderness in the right lower quadrant. The rest of her examination was otherwise unremarkable. A computed tomogram (CT) scan of the abdomen showed a large heterogeneous, partially necrotic abdominal-pelvic mass, 16.3 cm by 20.2 cm in size (Figure 1), and peritoneal carcinomatosis with small ascites. Chest CT scan (Figure 2) showed a moderate right-sided pleural effusion, as well as bilateral pulmonary nodules with mediastinal lymphadenopathy. Pertinent laboratory values on admission included potassium of 3.2 mg/dL, bicarbonate of 28.2 mg/dL, creatinine of 1 mg/dL, and phosphorus of 3.6 mg/dL.

Because of the size of her tumor, she was subsequently transferred to a tertiary health center for further evaluation and possible surgery. As seen in Table 1, the patient on admission was found to have hyperkalemia (potassium 6.2 mg/dL), hyperphosphatemia (phosphate 9.8 mg/dL), hyperuricemia (uric acid 15.1 mg/dL), and hypocalcemia (ionized calcium of 1 mmol/L), without having received any chemotherapy for her malignancy. She additionally went into renal failure (creatinine of 3 mg/dL) and severe metabolic acidosis (bicarbonate of 9 mg/dL with an arterial pH of 7.259). She also had a lactate of 11.1 mg/dL. LDH was also elevated at 1263 U/L, roughly six times the upper limit of normal. Based on these abnormalities, laboratory TLS was diagnosed; given her renal failure, she also had evidence of clinical TLS.

The patient's electrolytes worsened, with potassium of 6.5 mg/dL, phosphorus of 11.1 mg/dL, and a uric acid of 16.1 mg/dL. She also had a lactic acid level of 11.1 mg/dL. She was transferred to the ICU due to hypotension (blood pressure of 62/49 mm Hg), requiring vasopressor support, and increased work of breathing due to her profound acidosis (for which she was placed on rescue noninvasive bilevel positive airway pressure). Her renal failure was treated with intravenous normal saline (her creatinine eventually trended down to 2.3 mg/dL) while her hyperuricemia was treated with rasburicase 6 mg (uric acid trended down to 7 mg/dL). She was additionally started on a sodium bicarbonate infusion for severe metabolic acidosis (bicarbonate improved to 17 mg/dL). She was given kayexalate, D50, insulin, and calcium gluconate for her hyperkalemia, which eventually corrected to 4.3 mg/dL. LDH and lactic acid trended down to 1066 mg/dL and 5.6 mg/dL, respectively.

Final pathology from the biopsies of the abdominal mass and lung nodules taken at the community hospital was consistent with leiomyosarcoma with metastases to the lungs. The patient however was deemed a poor surgical candidate for debulking as well as a poor candidate for dialysis. She was additionally unable to be weaned off of vasopressor support. Given the patient's poor prognosis, the patient opted for comfort care. She was placed on hospice and expired subsequently.

## 3. Discussion

The Cairo-Bishop laboratory criteria of TLS, established in 2004, define laboratory TLS as meeting two of the following: uric acid > 8 mg/dL, potassium > 6.5 mg/dL, phosphorus > 4.5 mg/dL in adults (or a 25% increase from baseline in all), and calcium < 7 mg/dL (or a 25% decrease from baseline) [4]. These criteria were created in the setting of chemotherapy use (three days before chemotherapy or seven days after chemotherapy) as well as adequate hydration and use of a uric acid lowering agent. In addition, clinical TLS was defined as laboratory TLS as mentioned above plus creatinine > 1.5 times the upper limit of normal, oliguria cardiac arrhythmias, neuromuscular irritability, seizures, and sudden death [4].

TLS primarily occurs after initiating chemotherapy in hematologic malignancies. Aggressive non-Hodgkin's lymphoma (such as Burkitt's lymphoma), acute myeloid leukemia, and acute lymphoblastic leukemia have the highest risk of TLS [3, 4]. TLS develops because of rapid cell breakdown with chemotherapy [5]. TLS is rare in solid tumors unless they are large bulky tumors and/or they are highly chemosensitive.

According to a risk-stratification system, for TLS, high-risk group was defined as >5% risk for developing TLS [5]. This group consisted of hematologic malignancies such as Burkitt's lymphoma, diffuse B-celll lymphoma; ALL with WBC > 100,000/microL. Intermediate risk group (risk 1–5% for developing TLS) includes hematologic malignancies such as ALL with WBC < 100,000/microL, early lymphoblastic lymphoma, chronic lymphocytic leukemia, and bulky chemosensitive tumors such as germ cell tumors and small cell lung cancer. Most solid tumors are considered low risk (risk of TLS < 1%). Other malignancies at low risk for TLS include multiple myeloma, chronic myeloid leukemia, and acute myeloid leukemia with WBC < 25,000/microL [5].

Prophylactic uric acid lowering therapy is important in the prevention of TLS in malignancies that have the potential to develop it. Intravenous hydration promotes the excretion of uric acid; additionally, allopurinol blocks the production of uric acid by inhibiting xanthine oxidase. In malignancies with a high risk of developing TLS, aggressive intravenous hydration as well as rasburicase (although controversial for prophylactic use), which converts uric acid to a readily excreted metabolite, should be given prophylactically [5]. For intermediate risk patients, allopurinol should be used. For patients at low risk for TLS, IV hydration should be administered for prevention of TLS; administration of prophylactic uric acid lowering therapy is not recommended [5, 6].

Intravenous hydration and correction of other electrolyte derangements such as hyperkalemia are crucial in the management for TLS. Correction of hyperkalemia can be achieved via kayexalate as well as dialysis [3]. IV calcium should not be administered, however, to patients with hyperphosphatemia, because it promotes calcium phosphate precipitates [5]. A patient who does not respond to these measures may need renal replacement therapy to manage electrolyte abnormalities and renal failure [3, 5]. The mechanism behind renal failure can be attributed to calcium phosphate precipitation as well as to uric acid crystallization that occurs at the level of the renal tubules. Rasburicase is often recommended if there is evidence of renal failure and/or if there is a persistently rising uric acid level (typically if greater than 10 mg/dL). Often, when prognosis is poor, a palliative approach is suggested [7, 8].

Spontaneous tumor lysis syndrome makes up for only 15% of TLS cases and is typically seen in highly aggressive hematologic malignancies [8, 9]. There have been previous case reports of spontaneous TLS in solid tumors such as in lung cancers [10, 11], breast cancers [12, 13], germ cell tumors [14, 15], and prostate cancer [16]. However the prevalence is difficult to determine [16].

Additionally, TLS occurring in vaginal, vulvar, and ovarian cancers has been previously reported [7, 17]. However, those cases were related to the initiation of chemotherapy. Based on review of the literature, this is the only case of uterine leiomyosarcoma, where tumor lysis occurred spontaneously without the patient receiving chemotherapy.

The mechanism of occurrence of spontaneous TLS is hypothesized to be the consequence of necrosis developing in a large tumor as vascular supply is compromised by tumor growth and necrosis of tumor cells progress to tumor lysis from necrotic tumor cell contents being released into circulation [8]. The existing cases in literature including the current case indicate that, in solid tumors, tumor burden, especially with tumors that are highly proliferative and/or have areas of necrosis, may be a risk factor for the development of spontaneous TLS [10]. In the case of the patient mentioned above, it is likely her tumor burden with areas of necrosis that predisposed her to develop spontaneous TLS. This case highlights recognition of tumor lysis syndrome even with ordinary abdominal symptoms in the setting of metabolic derangements.

## 4. Conclusion

This case illustrates a presentation of spontaneous TLS in a gynecologic solid tumor which has not been previously reported. This case demonstrates that TLS may occur in large solid tumors even without the initiation of chemotherapy. Patients with large tumors should be closely monitored for metabolic derangements that may indicate the development of spontaneous TLS.

## Figures and Tables

**Table 1:** Progression of the patient's electrolyte derangements.

Patient disposition	Potassium (mg/dL)	Phosphorus (mg/dL)	Ionized calcium (mmol/L)	Uric acid (mg/dL)	Creatinine (mg/dL)	Bicarbonate (mg/dL)	LDH (mg/dL)	Lactate (mmol/L)
Admission to community hospital	3.2	3.6	N/A	N/A	1	28.2	774	3.8
Prior to transfer to tertiary care center	6.2	9.8	N/A	15.1	2.3	19.4	N/A	2.3
Admission to tertiary care center	6.2	9.8	1	15.1	3	9	N/A	11.1
On ICU admission	6.5	11.5	1.07	16.1	2.8	14	1243	5.5
Prior to death	4.3	6.5	1.14	7	2.3	17	1066	5
